# “Sharing in hopes and worries”—a qualitative analysis of the delivery of compassionate care in palliative care and oncology at end of life

**DOI:** 10.1080/17482631.2019.1622355

**Published:** 2019-06-17

**Authors:** Sarah Bessen, Raina H. Jain, W. Blair Brooks, Manish Mishra

**Affiliations:** a Geisel School of Medicine at Dartmouth, Hanover, NH, USA; b The Dartmouth Institute for Health Policy and Clinical Practice, Lebanon, NH, USA

**Keywords:** Compassionate care, end of life, palliative care, oncology, compassion fatigue

## Abstract

**Purpose**: To explore the methods through which physicians deliver compassionate care during end-of-life (EOL). Compassionate care provides benefits to patients and providers and is particularly important for patients with serious illnesses, yet its practice remains limited. We aim to qualitatively characterize methods utilized by physicians that facilitate the delivery of compassionate care at EOL.

**Methods**: We conducted 13 semi-structured interviews with physicians from palliative care and medical oncology subspecialities at a rural academic medical centre in New Hampshire. Interviews were transcribed and analysed using a qualitative research design.

**Results**: Participants described methods of compassionate care ranging from symptom control to less tangible, non-verbal methods. Primary barriers to the delivery of compassionate care were described as within the broader healthcare system and within the inherent emotional difficulty of EOL care. Physicians from both subspecialities emphasized the importance of successful inter-provider relationships.

**Conclusions:** Methods for delivering compassionate care at EOL are wide ranging, but barriers on a systemic and individual level should be addressed to make its practice more widespread. This can be accomplished, in part, by the standardization of EOL conversations, training physicians how to have meaningful EOL conversations, and integration of such conversations into electronic medical records.

## Introduction

The recent movement around patient-centred care in medical practice has been accompanied by an emphasis placed on the integration of compassion as part of standard medical care. Compassionate care, defined by the Schwartz Center for Compassionate Healthcare as that which “addresses the emotional and psychosocial aspects of the patient experience,” improves patient–provider relationships and has been shown to have tangible benefits to patients, providers, and institutions alike (Schwartz Center for Compassionate Healthcare, 2015). Institutions committed to compassionate care have lower staff turnover, have higher retention, are able to recruit more qualified staff, and have improved patient loyalty, while their patients also have shorter lengths of stay, lower rates of re-hospitalization, and fewer costly procedures (Schwartz Center for Compassionate Healthcare, 2015). Furthermore, empathy and effective communication, two major components of compassionate care, have been linked to improved outcomes across an array of conditions, including diabetes, cancer, and pain control, in addition to increased patient adherence to their clinical treatment (Lown, Rosen, & Marttila, 2011).

Compassionate care is particularly important for patients with advanced disease, given the emotional difficulty of receiving a poor prognosis, worsening symptom burden, and the vulnerability associated with confronting death and dying (Greer, Jackson, & Meier et al., 2013; Palfrey, Armour, & Grubb et al., 2016). Given the sensitive nature of the end-of-life (EOL) care, it is critical to understand the preferences of patients and their caregivers, as well as tailor EOL conversations accordingly (Parker, Clayton, & Hancock et al., 2007).

While the majority of patients and providers believe compassionate care is important for successful medical treatment, there is dissonance between patient and provider beliefs on whether compassionate care is delivered in practice (Schwartz Center for Compassionate Healthcare, 2015). Various studies have explored EOL care by surveying patients, but few have used an open-ended qualitative approach to understand how providers working with terminally ill populations exercise compassion in practice (Lendon, Ahluwalia, & Walling et al., 2015). Our aim in this study was to qualitatively characterize approaches used by palliative care physicians and medical oncologists to deliver compassionate care during EOL.

## Materials and methods

We conducted semi-structured interviews with 13 physicians (Table I) and elected for a qualitative approach in order to explore the nuanced ways in which physicians practice compassion. Providers from palliative care and medical oncology subspecialities were selected, given their frequent and often-overlapping interaction with terminally ill patient populations. An initial interview guide was created based on our primary research question, focusing on how palliative care physicians and medical oncologists operationalize compassionate care within the context of EOL care and conversations. The interview guide (see Appendix) was informed by a preliminary literature search and iterated throughout the study.

**Table I:** Participant characteristics

	(*n* = 13)
Gender, *n*	
Male	7 (53.8%)
Female	6 (46.2%)
Speciality, *n*(%)	
Palliative care	6 (46.2%)
Hematology/oncology	7 (53.8%)
Years in practice, median (range)^a^	10 (0.3, 30)
%1–1year	2
1–5 years	1
5–10 years	3
10+ years	7

^a^One provider completed both medical oncology and palliative care fellowships, but currently practices as a palliative care physician. This provider’s years in practice as a palliative care physician are considered in this table.

The study received exempt determination from the Committee for the Protection of Human Subjects, the Institutional Review Board at Dartmouth College. Study participants were physicians affiliated with the Palliative Care and Hematology/Oncology departments at Dartmouth-Hitchcock Medical Center, a 396-bed rural academic medical centre in Hanover, New Hampshire. Participants were recruited with an introductory e-mail including information regarding the goals of the project, with 8 of 9 palliative care physicians and 5 of 17 medical oncologists electing to participate.

During each session, one researcher guided the interview, while a second operated the audio recorder and took thematic notes. Each interview lasted 20–50 min. Audio-recordings were transcribed, and a thematic analysis was used to explore the similarities and differences among palliative care and medical oncologists, as well as to explore the dynamic between the two subspecialities. The interview guide was used to generate a code list, and codes were subsequently used to label study-relevant text segments. Each transcript was coded by two reviewers and later reviewed for agreement and to construct the primary themes.

## Results

Phenomenological research methods were used to consolidate the data from interviews with physicians from both disciplines into four key themes—(1) the importance of responding to patients’ physical and emotional needs, (2) the use of EOL conversations to empower patients, (3) systemic and emotional challenges that pose as barriers to the optimal delivery of compassionate care, and (4) the role of inter-provider relationships in the delivery of compassionate care.

### 

*1. Responding to patients’ physical and emotional needs*

Several participants commented that their techniques of delivering compassionate care involved tangible skills related to symptom control. Notably, oncologists frequently emphasized that palliative care physicians had a unique skill set to aid in the management of patients’ physical symptoms.

[Palliative care physicians] do spend more time, they are generally better at a lot of symptom management things, especially if it gets complex. So if it’s someone who has a lot of pain control needs, especially if they also have an addiction history, it can be very complicated. So they definitely have a skill set that’s different. (P1)

However, several participants stated that various intangible methods, such as conveying long-term presence as a provider and offering longitudinal support to the patient, were also critical to the delivery of compassionate care. A few participants also mentioned that non-verbal gestures such as hugging and hand-holding were helpful ways to address patients’ emotional needs.

There [are] a lot of like technical things–the skills of naming what they’re saying and validating their concerns and exploring more as they give you a piece of information … And then there’s the non-technical or I guess maybe less tangible things. So the nonverbal things, body positioning … I’m usually sitting or … I’m even kneeling on the floor, holding a patient’s hand if that’s appropriate. And sometimes it’s clear that people don’t want that kind of contact, but sometimes they do, and so I think also the skill of reading people and trying to get a sense of what they need from me, and then providing that to the best of my ability. (P2)

### 

*2. Using EOL conversations for patient empowerment*

EOL conversations were an important point of discussion during interviews, with providers emphasizing that such conversations should occur early and often with patients. The timing and frequency of EOL conversations allowed providers both to mirror and to accommodate the dynamic nature of the chronic disease and understand evolving patient priorities that could inform aggressiveness of care.

The reason I think that it is important to begin [EOL conversations] early is that patients need to assimilate this tough information over time, and if you try to slam it all on them at the very end, it’s overwhelming … I think allowing patients to titrate the amount of information they get over a long period of time is a far better way to do it. … that should begin as early as possible when you know you have an unfixable disease. (P3)

Providers also mentioned the use of EOL conversations to empower patients by educating them about their disease trajectory in a clear, concise manner. Importantly, several providers noted that they attempt to tailor the depth of the conversation to how much the patient wants to know about their prognosis.

What I hope to achieve is to empower these patients to the extent that they want to be, to know what they need to know about their disease, to make the best choices about that disease if they have complications. So, my goal is to sort of learn, “Who are you? What’s important to you? Who’s important to you? What are the things that frighten you?” (P3)

Participants, especially palliative care providers, often expressed the importance of “sharing in a patient’s hopes and worries” during EOL conversations in order to gain an understanding of what patients want to accomplish in their final days.

When you get into a conversation with a patient and their family … you try to delve into their hopes, their fears, their priorities … and the priority could be time—“I want more time on this Earth. I want more quality of life. I want less symptoms.” I mean you name it. There’s so many different things and how do we accomplish that? (P4)

Various participants also commented on the importance of having honest conversations to help patients have realistic expectations and understanding of the risks involved in their elected care plans.

If you are going to ask patients to tell you what is important to them … you need to put some kind of boundaries around what’s possible. You shouldn’t ask a patient “What do you want?” without making clear what actually is achievable. If you haven’t given someone some kind of prognostic framework, and then you say “Tell me what’s important,” they may say “I want to be cured” when that’s not an option. So I do think a critical part of these conversations is orienting a patient to what’s medically possible. (P3)

### 

*3. Addressing barriers to delivery of compassionate care*

When asked about barriers to successful EOL conversations, four primary themes emerged across both subspecialities: (1) the emotional difficulty inherent to having such conversations, (2) broader challenges associated with the healthcare system, (3) patients’ receptiveness to having an open, honest conversation regarding EOL priorities, and (4) compassion fatigue amongst providers treating terminally ill patients.

#### Emotional difficulty

Participants often noted the emotional difficulty of conversations surrounding EOL care for both patients and providers as a major barrier to successful EOL conversations.

I think clinicians don’t know how to have these conversations, they don’t feel comfortable. I think people have their own emotional experiences when they’re taking care of people who are really sick and they don’t know how to manage that, and so they avoid having those conversations because they don’t want to experience that emotional stuff. (P2)

#### Systemic challenges

A majority of participants also cited issues with the health-care system at large, namely, a lack of adequate time to interact with patients. Several participants commented that the shortage of time creates a difficult environment to communicate the necessary information to patients in an appropriate and considerate manner. Interestingly, a few participants commented that this issue could be addressed by standardizing the questions and content of EOL conversations, as well as by integrating such conversations into electronic medical records (EMRs).

I think the biggest barrier to these kinds of conversations … For other clinicians, big barriers include time. They suspect that this conversation will take a long time. Part of the reason for [using a standardized] conversation guide is to help them feel like they have the language so they can actually feel more confident and move through more quickly than they might otherwise … And I think another barrier really is that sometimes clinicians feel, “Well, I could go through all this work, but then it’s just going to get buried in my note and nobody’s ever going to see it. And so what’s the point right?” … We need systems so that it’s easy for these conversations to live and be visible. (P3)

#### Patient receptiveness

Furthermore, participants described these conversations as particularly difficult for providers without the specific training to engage with patients unwilling or unready to accept a prognosis.

If someone who knows they have an incurable cancer says “I really still want to go for cure” then I think I would say “I wish that was possible,” which is a little bit stronger than “I worry that a cure is not possible.” So it’s a way of being compassionate but also not preserving hope when it’s something that you know isn’t achievable. I think a lot of times doctors have trouble with that because they don’t want to take away hope. (P5)

#### Compassion fatigue

A majority of participants acknowledged that they themselves or their colleagues have experienced compassion fatigue and acknowledged it as a pertinent issue in medicine, especially within specialities interacting with terminally ill patient populations.

It’s something that … is a risk when you’re in a field where there’s a lot of suffering and sadness, and so you have to be prepared to be clear on what’s yours and what’s somebody else’s. So being with someone in their suffering isn’t necessarily taking that all on yourself and experiencing it yourself. I sometimes use the analogy of being like a sponge where I can be with someone and sort of absorb their experience of illness, but at the end of the encounter, then I have to squeeze myself out. (P5)

When asked about methods to prevent compassion fatigue, palliative care physicians particularly emphasized the importance of having colleagues to share experiences and talk openly with.

I feel like it is true that some providers can become desensitized just based on the volume of work they’re expected to do … I think that we’re pretty lucky in palliative care that we get longer time with patients and I think that we have built in ways of maintaining resilience and recognizing when something has become just too difficult and we need the help of our peers … and I think there might be less support for the hospitalist. I think for us it’s built into our job that we can go talk to each other about challenging cases and cry together and remember patients together. (P6)

Participants also spoke of the importance of self-awareness and the ability to maintain boundaries in order to prevent overidentification with patients.

There’s some patients that that are higher risk for boundary violations or permeability. So in palliative care, we pay attention to trying to understand which patients do that for us, and often not surprisingly, it’s often different patients for different people. It often tends to be someone that’s similar to you. So, we try to do some triage and … also just recognizing this is that for me, so I need to be particularly careful and attentive to the boundaries. (P6)

### 

*4. Importance of collaborative inter-provider relationships*

Participants across both palliative care and hematology/oncology specialities commented that interprofessional teamwork allowed for greater delineation of provider responsibilities, which could help alleviate limitations posed by time burden and inform patient decisions regarding who to seek out for clinical versus emotional care.

There’s some patients that have challenging pain issues and/or symptom issues and palliative care can really use their expertise to manage those … sometimes for the challenging drug patient, like somebody [with a] history of abuse or views or overuse, having palliative deal with their narcotics frees us from sometimes a difficult conversation and it can keep the oncologist-patient interaction as a more positive one … We [medical oncologists] can sort of have our conversation about CT scans, but you’re not doing both things, so that’s helpful. (P7)

However, various participants described persistent misconceptions among both patients and providers regarding the role of palliative care. Specifically, the primary association of palliative care providers with death prevents patient and provider receptivity and potentially delays referrals that could improve compassionate care at EOL.

I think a lot of people see [palliative care physicians] as the death people. Certainly nationally [palliative care physicians] get called way too late because people associate us with dying patients only and don’t recognize that actually our greatest benefit is probably way upstream. [It] changes the whole experience of an illness for a patient and the providers. (P2)

## Discussion

In this study of the delivery of compassionate care at EOL, several common threads were identified during conversations with both palliative care and medical oncologists regarding compassionate care at EOL.

Amongst the interviewees in our sample, participants from both subspecialities discussed the importance of combining both tangible methods, such as symptom control, and intangible or non-verbal methods as methods of delivering compassion. EOL conversations were named as a potential space for patient empowerment, and participants widely pointed to the importance of collaboration among providers to optimize care. Lastly, participants described primary barriers to compassionate care arising from within the healthcare system as well as within the inherent emotional difficulty of EOL care. Moving forward, several steps can be taken to address such barriers and improve the delivery of compassionate care to terminally ill patients.

An emphasis on patient–provider relationships and shared-decision-making is especially critical within the context of patients suffering from terminal diseases, given wide variation in patient priorities (Bélanger, Rodríguez, & Groleau et al., 2014; Steinhauser, Christakis, & Clipp et al., 2000). However, various studies have demonstrated that tendencies to delay these conversations or focus on curative measures impede decision-making opportunities and compromise clinical outcomes (Bélanger et al., 2014; Galushko, Romotzky, & Voltz, 2012; Hak, Koëter, & van der Wal, 2000; Institute of Medicine, 2015; Quirt, Mackillop, & Ginsburg et al., 1997). A study by Belanger et al. found that the organization of care, for example, in the timing of referrals to palliative care, shaped whether patients were able to participate in decision-making, with earlier referrals influencing remaining decisions as well as patients’ prognostic awareness (2014).

Our study illuminated the need for a broader cultural change surrounding EOL care and conversations in order to address delayed referrals. The present findings emphasize that the emotional difficulty in confronting EOL is multidimensional, as the discomfort poses a barrier for both providers and patients. From the provider perspective, it remains important to normalize such conversations to reduce the accompanying hesitation in future conversations and to be able to have an honest, productive dialogue about patients’ priorities during the time they have remaining. This need has been noted in previous literature, especially concerning advance directives (Bélanger et al., 2014), a topic closely aligned with EOL conversations. A 2015 report from the Institute of Medicine emphasized the importance of public education and engagement efforts with people from diverse communities in order to normalize difficult conversations and provide the tools needed to actively participate in conversations about death and dying (2015). One component of such a change may also entail a cultural shift surrounding the perceived role of palliative care physicians, who in our study, often stated that their mischaracterization as “death people” led to delayed referrals and potentially decreased the quality of patient care. These efforts remain critical so that more patients can receive the care they desire near the EOL.

Furthermore, the standardization of EOL conversations would directly aid in normalizing the care of patients facing the latter stages of disease. Standardized tools that equip providers with patient-tested language, such as the *Serious Illness Conversation Guide* from Ariadne Labs, should serve as a primary reference or component of training efforts in decision-making conversations (Bernacki & Block, 2014). Standardizing tools can help promote effective, efficient conversations regarding EOL priorities and alleviate systemic concerns that were voiced during interviews, specifically pertaining to the lack of time to conduct meaningful conversations. Such tools would also address concerns surrounding the emotional difficulty of EOL conversations while ensuring that patients’ needs are comprehensively addressed.

While these types of tools provide a strong framework for EOL conversations, a more standardized training process may also help providers across different disciplines feel more comfortable initiating and having difficult conversations. Of all the interviews conducted with providers, only palliative care physicians stated they previously had formal training in EOL conversations, while none of the medical oncologists encountered such training as a component of their residency or fellowship. Instead, the majority credited their ability to have such conversations to their experiences (both positive and negative) observing their mentors. Our study findings strongly support prior findings (Gelband, 2001; Institute of Medicine, 2015; Parikh, White, & Buckingham et al., 2017) regarding the importance of educational institutions and healthcare delivery organizations prioritizing the appropriate training of clinicians across all specialities that care for individuals with advanced illnesses.

EOL care can be even further be standardized through the integration of such conversations into the EMR system of each institution, a suggestion that was echoed in an interview with a palliative care physician. The results of each conversation would then be easily accessible for future visits and other providers, should care ever be transferred between physicians. Collectively, such efforts to standardize care can be effective in improving EOL discussions and compassionate care. Most importantly, these efforts can also enable clinicians to develop the skills necessary to conduct highly individualized conversations with patients.

Lastly, various studies have identified potential predictors for compassion fatigue, including emotional depletion, distress from clinical situations and co-workers, and time pressure (Kleiner & Wallace, 2017; Weintraub, Geithner, & Stroustrup et al., 2016; Zwack & Schweitzer, 2013). Providers in the present study also spoke of compassion fatigue as a relevant phenomenon based on either their own experiences or those of their colleagues. Participants described the importance of self-awareness, the ability to maintain boundaries, and the ability to focus on meaningful patient experiences and reframe goals of care towards achievable outcomes. Palliative care physicians, in particular, discussed the importance of having built-in time dedicated to sharing patient experiences with co-workers, especially given the highly interdisciplinary nature of their work which often involves a combination of palliative care physicians, other healthcare providers, and social workers. In contrast, medical oncologists largely focused on individual-based strategies to prevent compassion fatigue (e.g., maintaining self-awareness and boundaries).

It should be noted that while both physicians within palliative care and medical oncology often interact with terminally ill patients, physician reflections in the present study were not focused on a specific patient population. Furthermore, these clinical experiences represent those of a single rural academic center with a relatively small sample size and thus may not be generalizable. Nevertheless, our study findings illuminate wide-ranging methods for delivering compassionate care at the EOL, as well as systemic barriers, such as time pressure and patient caseloads, that prevent this practice among physicians. As such, it remains necessary to address these barriers through the standardization and assimilation of EOL conversations as well as the facilitation of an environment where providers can dedicate time to team-based and individual resilience strategies.

## Appendix Appendix

### Interview guide

1. How would you describe your patient population?

2. At what stage of disease do you normally first see your patients?

3. How long have you been in practice?

4. Have you had specific training in end-of-life care, and if so, how?

5. How do you define compassion?

6. How do practice compassion? How do you practice compassion during end-of-life (EOL)?

7. At what point do you feel it is appropriate to have a conversation about EOL priorities? How do you define end-of-life?

8. What questions do you ask patients during EOL conversations?

9. What are your goals with EOL conversations? What do you want to achieve by having this conversation?

10. What is important for providers to convey to patients during EOL conversations?

11. When a patient’s priorities do not align with your medical recommendations, or a patient’s goals are not medically appropriate, what is your approach going forward?

12. What do you perceive as barriers to having successful EOL conversations?

13. Do you feel that it has become harder to remain compassionate over the course of your career (i.e., compassion fatigue)? Do you feel that providers become desensitized to patients’ experiences?
